# Design of a Unique Device for Residual Stresses Quantification by the Drilling Method Combining the PhotoStress and Digital Image Correlation

**DOI:** 10.3390/ma14020314

**Published:** 2021-01-09

**Authors:** Miroslav Pástor, Martin Hagara, Ivan Virgala, Adam Kal’avský, Alžbeta Sapietová, Lenka Hagarová

**Affiliations:** 1 Department of Applied Mechanics and Mechanical Engineering, Faculty of Mechanical Engineering, Technical University of Košice, Letná 9, 04200 Košice, Slovakia; miroslav.pastor@tuke.sk (M.P.); martin.hagara@tuke.sk (M.H.); adam.kalavsky@tuke.sk (A.K.); 2 Department of Mechatronics, Faculty of Mechanical Engineering, Technical University of Košice, Park Komenského 8, 04200 Košice, Slovakia; 3 Department of Applied Mechanics, Faculty of Mechanical Engineering, University of Žilina, Univerzitná 8215/1, 01026 Žilina, Slovakia; alzbeta.sapietova@fstroj.uniza.sk; 4 Institute of Geotechnics of Slovak Academy of Sciences, Watsonova 45, 04001 Košice, Slovakia; hagarova@saske.sk

**Keywords:** residual stresses, hole-drilling, PhotoStress, digital image correlation, experimental analysis, finite element analysis

## Abstract

This paper presents a uniquely designed device combining the hole-drilling technique with two optical systems based on the PhotoStress and digital image correlation (DIC) method, where the digital image correlation system moves with the cutting tool. The authors aimed to verify whether the accuracy of the drilled hole according to ASTM E837-13a standard and the positioning accuracy of the device were sufficient to achieve accurate results. The experimental testing was performed on a thin specimen made from strain sensitive coating PS-1D, which allowed comparison of the results obtained by both methods. Although application of the PhotoStress method allows analysis of the strains at the edge of the cut hole, it requires a lot of experimenter’s practical skills to assess the results correctly. On the other hand, the DIC method allows digital processing of the measured data. However, the problem is not only to determine the data at the edge of the hole, the results also significantly depend on the smoothing levels used. The quantitative comparison of the results obtained was performed using finite element analysis.

## 1. Introduction

Residual stresses are present in almost all materials. They may arise during the manufacturing process or over the life of a material. Their quantification process is essential because their superposition with stresses occurring in the material due to the external loading can lead to material failure. The measurement techniques used for residual stress quantification can be divided into three main groups: non-destructive, semi-destructive, and destructive.

In a production environment, determination of residual stresses in the material is usually based on the standardized hole-drilling strain gauge method allowing identification of in-plane residual stresses near the measured specimen surface made from an isotropic linear-elastic material.

The methodology of residual stresses quantification using the hole-drilling strain gauge method involves several steps:

1. smoothing of the specimen surface using, e.g., chemical etching (manufacturing technologies such as abrading or grinding have to be avoided);
2. attaching a strain gauge rosette with special measuring grids to the examined location;
3. drilling a through-hole at the geometric center of the strain gauge rosette in one step to the specimen whose thickness is much less than the diameter of the hole (denoted as thin) or a blind hole in a series of steps to the specimen whose thickness is much greater than the diameter of the hole (denoted as thick);
4. measuring the resulting relieved strains whose values depend on the residual stresses existing in the material of the hole;
5. determining the residual stresses in the removed material using mathematical relations based on linear elasticity theory.

Stresses that remain approximately constant along the depth are defined as uniform stresses. If the stresses vary significantly with depth, they are known as non-uniform stresses. If the residual stress in a thin specimen material is investigated, the uniform stress measurement is specified. Both uniform and non-uniform stress measurements are specified for thick specimens. The accuracy of the results is satisfying if the residual stresses quantified in a thick specimen material do not exceed about 80% of the material yield stress. On the other hand, the residual stresses determined in a thin specimen material are less than approximately 50% of the material yield stress. Experimental measurements described in the following parts of the paper will be applied to the investigation of strains relieved in the area surrounding the hole cut into the thin specimen loaded by uniaxial tension. Due to similarity, the methodology for residual stress quantification performed in a thin specimen, drilling a through-hole as described in detail in ASTM E837-13a standard [1], can be found in Appendix A.

Many commercial devices allow control of the hole or the core milling process and evaluate the results using their own software. Over recent years, some modifications in the milling process have occurred, too, e.g., translational motion of the milling cutter has been replaced by circular motion.

Measurement techniques such as moiré interferometry, electronic speckle pattern interferometry, shearography, photoelasticity as well as digital image correlation belong to the group of non-contact experimental methods, which gradually replace conventional strain gauges used for the strain analysis at a point (or its near surroundings) and provide full-field information about the strain distribution. Some of them have already been adapted for residual stress quantification.

The moiré interferometry (MI) method works on the principle of diffraction grating interferometry, where interference fringes occur after each deformation of the object surface. The grating can be physically attached or just virtually generated on the object surface by two symmetric beams of light transmitted from the same source of coherent laser emission. Key characteristics relating to the use of MI for residual stress analysis are high displacement and spatial resolution, the possibility for micro-scaled measurements and incremental hole-drilling investigation. Ya et al. [2] designed a unique measuring system based on phase-shifted MI combined with the hole-drilling method, and analyzed the residual stresses on an aluminum plate. The use of MI in the determination of residual stresses, e.g., in the AS10OU3NG material with one surface shot-peened [2], composite materials [3], welded titanium alloy plate [4] as well as a uniaxially tensioned plate made from black polymethylmethacrylate [5] suggest the possibility of using this method in a wide range of materials.

Electronic speckle-pattern interferometry (ESPI) is based on the interference of two monochromatic laser beams, i.e., a subject beam reflected from the object surface and a reference beam creating reference speckle-effect on the image plane of the CCD (charge-coupled device) camera. The method does not require to create grid or speckles, has a high displacement resolution [6,7], allows performing measurements outside the laboratory [8], and removes the effect of rigid-body motion [9,10]. Analysis can be done on curved and rough surfaces using the incremental hole-drilling method. A portable measuring system with one symmetrical dual-beam illumination constructed by Viotti et al. [11] is another specially designed device that provides automatic calculation of residual stresses from measured displacements using the least square method. In 2017, Lothhammer et al. [12] constructed a measuring device designed for the residual stress quantification on the resistance-welded pipes. According to the authors, their device provides a more effective way to quantify the non-uniform stress distribution obtained at the same quality level compared to the conventional techniques.

Digital image correlation (DIC) is based on the comparison of digital images captured during the loading of an analyzed object. It is not as sensitive to ambient vibrations as the MI or ESPI methods, and therefore it is suitable for use in a production environment. In all the following references, specially adapted devices combining DIC and the core- or hole-drilling technique were used to quantify residual stresses. In 2005, one of the first residual stresses measurements using DIC was carried out by McGinnis et al. [13], when the authors investigated steel plates and reached less than 7% error in normal stress, which established the robustness of 3D DIC to capture the expected displacements. In 2006, Nelson et al. [14] calculated the residual stresses from displacements using dimensionless relations derived from numerical analysis, and good correspondence with the results acquired by the holographic method was achieved. In 2008, a hole-drilling device with an integrated camera allowing measurement of displacements on the object surface of 7 × 5.6 mm was developed [15]. The accuracy of the device was tested by the analysis of compression residual stresses simulated on the pipe loaded by the testing machine, whereby the acquired results similarly correspond to the results obtained by strain gauge rosette. In 2017, Baldi and Bertolino [16] developed a low-cost residual stress measuring instrument with integrated digital image correlation (iDIC). Using the proposed approach, the rigid-body motion is easily compensated for and no decorrelation problem results from large translations. The use of 2D iDIC was described in [17], where Baldi carried out measurements on specimens made from orthotropic material. The obtained results confirm that his proposed device is as accurate as previously developed optical techniques, however, its measuring range is significantly larger. In 2017, Rief et al. [18] designed a 3D DIC system connected to the hole-drilling device by a rigid frame ensuring that there was no sideward movement of the drill during the drilling process. The advantage of their device is that it is pre-calibrated since the camera’s distance to the object surface is constant. In 2020, Brynk et al. [19] also used a fixed stereo-camera system placed symmetrically to the milling cutter attached to the mechanism allowing its movement in the direction perpendicular to the specimen surface and analyzed residual stresses in the LVM316 steel. The milling process was performed by a stepper motor and steered with an Arduino microcontroller allowing precise drilling of holes to desired depth as well as removing of the drilling head from the cameras’ observation field. The current research done by Babaeeian and Mohammadimehr [20] aims to analyze the influence of the time elapsed effect on the levels of quantified residual stresses in composites.

Quantitative comparison of the displacement resolution and disadvantages of the aforementioned methods are shown in Table 1.

In this paper, which is an extension of the conference contribution [21], the unique device combines hole-drilling with two optical methods and differs from the devices described previously in that the cameras of the correlation system (used as a tool for the full-field displacement/strain analysis) move with the cutting tool. As the digital image correlation method is based on the correlation of digital images, it was necessary to verify whether the achieved positioning accuracy of the proposed drilling device was sufficient for reliable strain analysis. To eliminate the human failure factor during the measurement process, control software for adjustment of several parameters was created. The most important parameters adjusted were the shift and velocity of the block to which the milling tool was attached.

The initial testing of the drilling device found undesired mechanical clearances, which could significantly influence the measured data and, therefore, the device’s design was improved several times. The results obtained from testing of the optimized prototype showed that the aforementioned technical problem was eliminated.

## 2. Development and Testing of the Unique Hole-Drilling Device

In the development of the unique drilling device, the authors used their own experiences obtained from using commercially produced devices SINT MTS 3000 (SINT Technology, Calenzano, Italy), SINT MTS 3000 Ring-Core (SINT Technology), and RS 200 (Vishay Precision Group, Malvern, PA, USA) not only in laboratory conditions but also by solving problems in technical practice [22,23,24]. The common feature of the aforementioned devices is adjusting the position of the milling cutter in the center of the strain gauge rosette. Positioning of milling cutter in RS200 and SINT MTS 3000 is performed manually using X-Y adjusting screws. In SINT MTS 3000 Ring-Core, semi-automatic positioning is undertaken because the center of the milled core is adjusted through a camera, which is not further used in the residual stresses quantification procedure. After drilling the hole or the core, its real position towards the center of the strain gauge rosette has to be determined. If eccentricity occurs, its value is entered into the evaluation software for the correction of the residual stresses calculation. Analysis of the eccentricity effect in the measurement of the hole-drilling residual stresses was carried out, e.g., by Ajovalasit [25], Beghini et al. [26] or Barsanti et al. [27]. As mentioned in the introduction, the topic of residual stresses determination using optical full-field methods in combination with the hole-drilling method is very up-to-date. Many authors devote themselves to develop their own drilling devices, where the optical system is mostly stationary, and the movement is undertaken by the milling cutter. There are two types of milling tool movements used—translational performed in the milling direction or a combination of translational and rotational movement.

**Figure 1:**
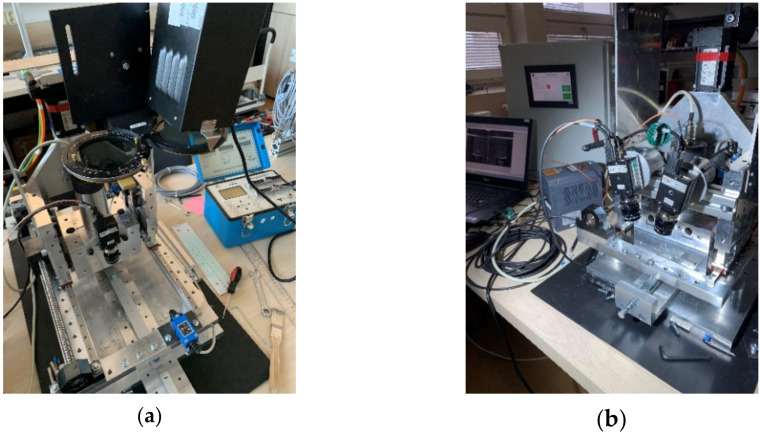
The prototype of the unique drilling device with: (**a**) a single-camera (2D) Q-400 digital image correlation (DIC) system and polariscope LF/Z-2; (**b**) a stereo-camera (3D) Q-400 DIC system.

In this paper, the design of such a drilling device is presented in which not only the milling tool moves to cut the hole, but also the optical system can be attached, used to determine displacement/strain fields, to the moving position (Figure 1). Achieving highly accurate positioning of the drilling device’s mechanical parts (like 10^−3^ mm) and desired repeatability of the measurements become crucial parameters required for the application of the above-mentioned methodology. Commercially produced drilling devices are also designed in such a way as to ensure the motion of their mechanical parts as accurately as possible.

In the design of the unique drilling device, the authors considered technical parameters of the optical systems used at the authors’ workplace, namely LF/Z-2 reflective polariscope (Vishay Precision Group, Malvern, PA, USA), as well as single-camera (2D) and stereo-camera (3D) digital image correlation system Q-400 (Dantec Dynamics A/S, Skovlunde, Denmark) (Figure 1).

### 2.1. Mechanical Design

When developing the drilling device, the authors were inspired by the construction of the SINT MTS 3000 Ring-Core system. However, the DIC system used in the designed device was, among other things, also used for the deformation analysis. The advantage of the strain analysis performed by the full-field optical methods is that the correction of the hole position carried out at the end of the measurement is unnecessary. If there occurs an insignificant change in the position of the drilled hole, ultimately it means that the deformation analysis will be made at a distance very close to the planned one. In the case of residual stresses determination, deviation from the analyzed location up to 1 mm does not cause a significant measurement error in the investigated stress levels. In respect of the experimental procedure, it is required to keep the essential principles valid for the commonly used hole-drilling method, e.g., regular changing of milling tools whose failure may lead to negative influenced results [28].

The designed mechanism allows motion in vertical and horizontal directions through the ball screws with pitch accuracy T5 (±0.023/300 mm). The ball screws are driven by B&R servomotors 8LVA23.B1015D100-0 controlled by servo driver 8EI4 × 5MWD10.0600-1. The servomotors have a nominal speed of 1500 rpm, a maximum speed of 6600 rpm, and a nominal torque of 1.33 Nm. The servomotors are connected to the ball screw by a flexible coupling. They use EnDat encoders with resolution 18/16 bits single turn/bits multi-turn, respectively. The pitch screw is 5 mm per round. The designed device uses a spindle with power of 850 W for drilling controlled by a frequency converter. It is possible to attach the single- or stereo-camera digital image correlation system to the lateral static part of the device, i.e., the camera will only move in the horizontal direction or to the block the milling tool is attached to. In such a case, the camera moves in two mutually perpendicular directions, i.e., horizontal and vertical.

### 2.2. Control System Design

**Figure 2:**
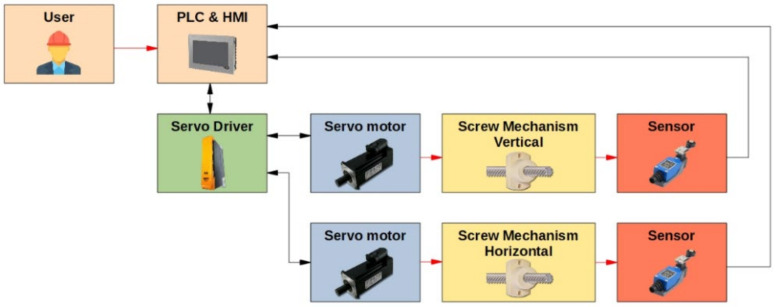
Information flow of control system.

The system is controlled by PLC 4PPC70.0702-20B with an implemented touch screen (power panel) for a HMI (human–machine interface). The flow of the information is shown in Figure 2. There are two working modes: manual and automatic. The manual mode allows the user to control the motion in both axes either by constant velocity motion or positioning the axis by setting the position value. This mode is used for the adjustment of the milling cutter with respect to the surface of the analyzed specimen. On the other hand, the automatic mode performs strictly predefined cycles of the drilling process defined by the ASTM E837-13a standard. In automatic mode, there are several parameters such as number of cycles, steps of drilling, required positions in both axes, delay after each cycle, velocity of drilling and the velocity of motion in both axes, which need to be set.

The algorithm of automatic mode is as follows (Algorithm 1).
**Algorithm 1** Automatic Mode1: Setting of the required drilling parameters 2: Motion to the reference position in vertical and horizontal axes 3: **WHILE** (actualCycle ≠ requiredCycles) 4:    Motion in vertical and horizontal axes upon the pattern 5:    **IF** (milling machine = turn off) THEN 6:         milling machine -> turn on 7:    **END_IF** 8:    **WHILE** (drillingTime ≠ requiredDrillingTime) 9:             Motion in y-axis: cycle*drillingStep 10:           Counting of drillingTime 11:    **END_WHILE** 12:    Motion in y-axis: -cycle*drillingStep 13:    Motion in y-axis and x-axis to reference position 14:    Delay after the end of the cycle 15:    cycle = cycle + 1 16: **END_WHILE** 17: milling machine -> turn off


### 2.3. Testing of the Positioning Accuracy

**Figure 3:**
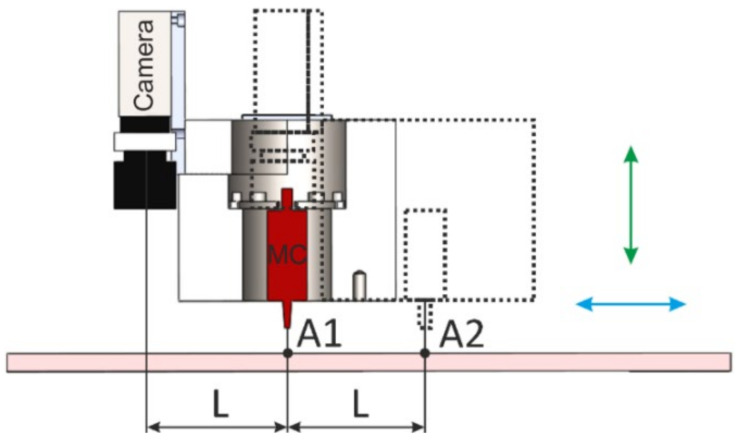
Principal scheme of the device positioning used for drilling of the hole and capturing digital images of the analyzed area by DIC in a series of steps.

The measurement process of the designed device with the DIC system is based on a mutual change in the position of the DIC system and the milling cutter. At first, the position of the milling cutter (MC) is adjusted to the analyzed area A1 of the specimen (see Figure 3). Subsequently, the milling tool is shifted to a new position A2, where distance L corresponds to the distance between the camera(s) and the axis of the milling cutter. In this position, the DIC system captures a reference image of the analyzed specimen area, after which the milling cutter is returned to A1 position, and the drilling process starts. After drilling the first step, the milling cutter moves to A2 position again, and the digital image corresponding to the first drilling cycle is captured. The process is repeated until the desired depth of the hole is achieved.

**Figure 4:**
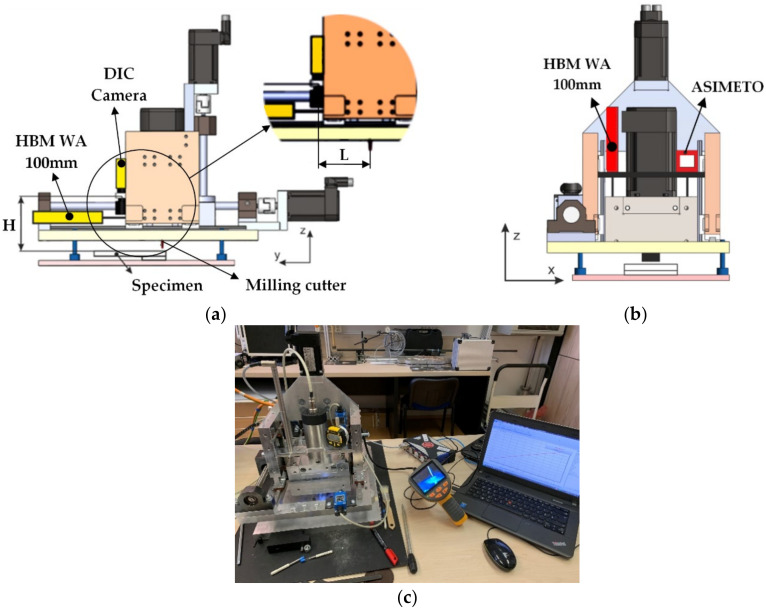
Testing of the positioning accuracy in: (**a**) horizontal direction; (**b**) vertical direction; (**c**) measuring string.

Several methods based on different physical principles were used for the validation of the device positioning accuracy. The testing aimed to verify the real change of the milling tool position adjusted by the control software (by servo driver). The experimental procedures were performed in two stages:

- testing of the horizontal positioning (Figure 4a),
- testing of the vertical positioning (Figure 4b).

**Figure 5:**
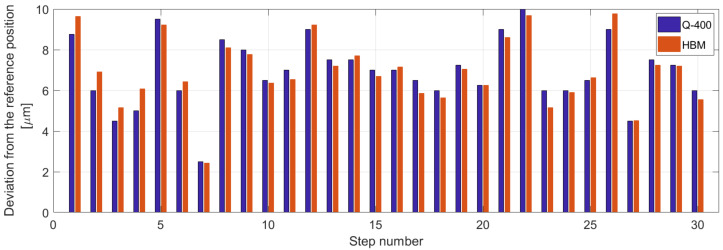
Deviations from the reference position of the device obtained for 30 repetitions of its movement to a new position at the distance 81 mm and subsequent return captured by the inductive displacement transducer WA-100 mm and 2D DIC.

The measurement in which the mutual position between the camera of the 2D DIC system and the milling cutter changed 30× was performed to obtain information about the accuracy and repeatability of the device positioning. The horizontal direction shift was adjusted to the value L = 81 mm corresponding to the distance between the lens of the 2D DIC system and the milling cutter axis (Figure 3). The results obtained, which showed high accuracy of the desired value and achieved repeatability, are given in Figure 5. The detailed analysis suggested that deviations from the reference device position (after its move to a new position at a distance of 81 mm adjusted by the control software and return) were 10^−3^ mm. The same graph shows that the deviations registered by the inductive displacement transducer WA-100 mm (HBM, Darmstadt, Germany) and 2D DIC system Q-400 (Dantec Dynamics A/S, Skovlunde, Denmark) are similar.

**Figure 6:**
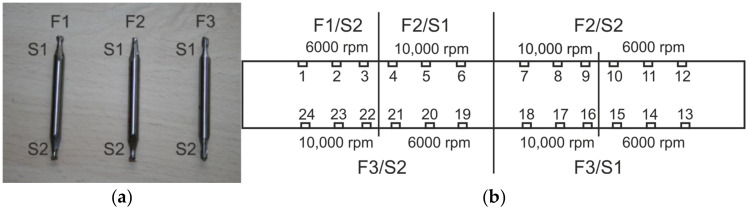
Testing of the positioning accuracy: (**a**) milling cutters F1, F2, F3 with a diameter of 3.2 mm and denotation of the sides; (**b**) location of the drilled blind holes.

Testing of the developed hole-drilling device positioning accuracy in a vertical direction, and analyzing the shape of the drilled hole were carried out on the specimen made from EN AW-5083 material, in which the blind holes were drilled using milling cutters provided with the RS 200 device, concretely the miller with a 3.2 mm tool diameter (Figure 6a). Measurements were performed according to the methodology set forth in the ASTM E837-13a standard, i.e., blind hole drilling up to 2 mm deep performed in 20 steps [1]. In this phase, all the steps were set up with the same increment of 0.1 mm (used to examine stresses uniformly distributed over the depth of the specimen).

For the measurement of the drilling device vertical shift, the inductive displacement transducer WA-100 mm and digital indicator ASIMETO series 405 (ASIMETO, Weissbach, Germany) were used (Figure 4c). In several cases, the above-mentioned transducers captured any higher deviations towards the value adjusted in the control software than in the horizontal direction (see Table 2).

**Figure 7:**
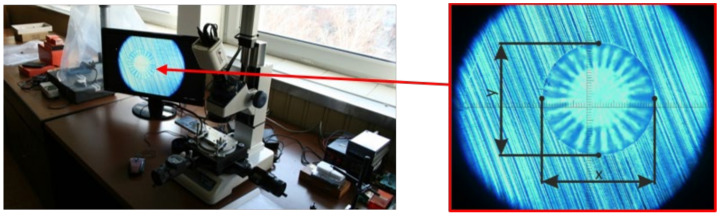
Measurement of the blind holes circularity in two mutually perpendicular directions.

The analysis of dimensions was carried out on a series of drilled blind holes (Figure 6b), whereby the influence of the cutting speed and the milling cutter wear was reviewed. It was experimentally proved that the developed drilling device can cut blind holes in accordance with the requirements specified in ASTM E837-13a standard. Examination of the shape of blind holes, i.e., analysis of their circularity achieved after the last (twentieth) drilling step and their cylindrical shape along the depth was undertaken using a specially adapted microscope TM-505B (Mitutoyo, Kanagawa, Japan), (Figure 7). The averaged distances of two antipodal points measured three-times in two mutually perpendicular directions are listed in Table 2.

**Figure 8:**
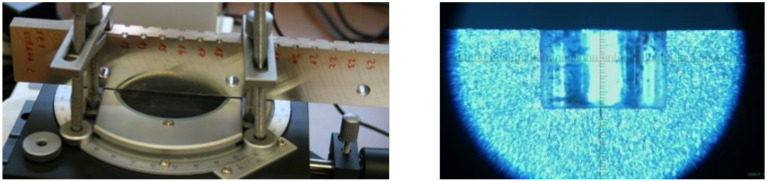
Measurement of the blind holes depth performed on the split specimen.

The circularity of the cut holes was validated by checking the cylindrical part shape of the blind holes carried out on a split specimen using a specially adapted microscope TM-505B (Figure 8). These measurements also provide information about the real depth of the drilled blind hole, which was set to 2 mm by the control software. Table 2 shows that the real depth (registered by microscope) of the drilled blind hole is 2.002 ± 0.0003 mm and, thus, the achieved accuracy is equal to the top commercially produced drilling devices.

It can be stated that if the control of the vertical shift will be realized by the inductive displacement transducer WA-100 mm, the real depth of the drilled blind hole could be assessed based on the results in Table 2. It has to be noted that the control measurements of the depth of the drilled hole using an electron microscope cannot be used for real structures.

## 3. Experimental Measurements

After performing testing measurements with satisfying results, the authors carried out analysis of the displacement/strain fields evaluated in the vicinity of the hole cut by the designed prototype of the drilling device into the testing specimen loaded by a known loading.

**Figure 9:**
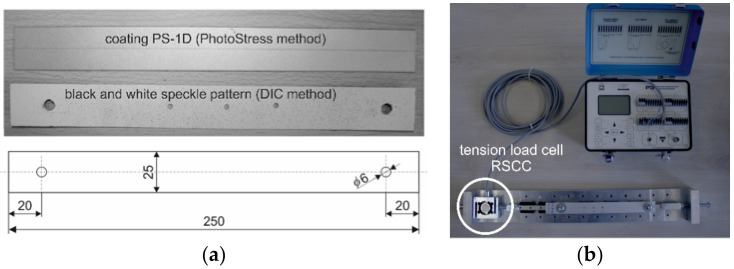
Testing specimen made from the PS-1D material: (**a**) the overall view with dimensions; (**b**) loading mechanism.

The full-field strain analysis was undertaken on the specimen made from the PS-1D material (Vishay Precision Group, Malvern, PA, USA) loaded by uniaxial tension loading (Figure 9a) registered by the RSCC-50 kg sensor (HBM, Darmstadt, Germany) (Figure 9b). The given mechanical/optical properties of the analyzed strain-sensitive plastic coating are: Young‘s modulus of elasticity E = 2500 MPa, Poisson’s ratio μ = 0.38, thickness t = 0.5 mm and the fringe value of coating f = 3790 μm/m/fringe. The dimensions of the specimen are given in Figure 9a. The through-hole lying on the specimen’s longitudinal axis was cut when the specimen was loaded by the uniaxial tension force of 250 N, and the relieved strains were observed using a polariscope based on the PhotoStress method as well as 3D DIC system. Measurement performed on any specimen in the near vicinity of the cut through-holes (due to the specimen’s thickness) was performed under the same cutting conditions (cutting speed and velocity of horizontal and vertical positioning). The advantage of such a measurement method is that the results in the form of the relieved strain fields obtained by two different optical methods are observed on the specimen made from the same material and can be further analyzed (compared).

### 3.1. PhotoStress Method

**Figure 10:**
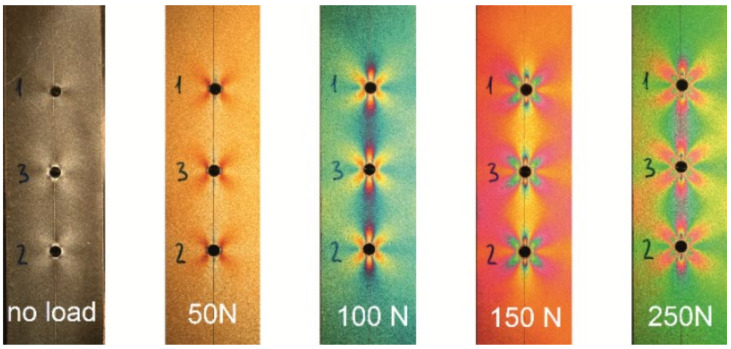
Characteristic change of the isochromatic fringes observed in PhotoStress method during increasing uniaxial loading.

The PhotoStress method is a non-contact full-field measurement technique used to determine surface strains and transform them into stresses occurring in a structure. To use this method, a special strain-sensitive plastic coating needs to be bonded to the analyzed structure. Subsequently, after applying the loading to the structure, its strains are transmitted to the coating assuming the same strain condition as the part of the structure, which it is bonded to. After illuminating the coating by polarized light emitted from a reflection polariscope, a colorful pattern that is viewed through the polariscope occurs due to strain. There are two types of color pattern investigated known as isoclinic and isochromatic fringes. At every point on an isoclinic, the directions of principal strains are parallel to the direction of the analyzer and polarizer polarization. The photoelastic strain pattern appears as a series of successive and contiguous, variously colored bands known as isochromatic fringes representing a different level of the principal stresses difference [29,30]. When the loading is applied to the structure, the coated part color changes from black characterizing the no-loading state of the structure and first colors appear in the areas of the highest stress. After increasing the loading, the color fringes spread throughout the coated part (Figure 10), additional fringes are generated in the highly stressed areas of the investigated structure and move towards the regions of zero or low stress until the maximum load is achieved.

The quantitative analysis of principal strain difference (strain intensity) occurring at any point of the coating can be quickly performed using a digital compensator attached to the polariscope. Strain intensity analysis depends only on the recognition of the fringe order defined by a color and understanding of the relationship between the fringe order and strain intensity as follows:

(1) $$\varepsilon_{1}-\varepsilon_{2}=N\cdot \frac{\lambda }{2\cdot t_{c}\cdot K}=N\cdot f$$

where $\varepsilon_{1},\varepsilon_{2}$ are the principal strains, N is the fringe order, λ is the wavelength of the light emitted by polariscope, $t_{c}$ is the thickness of the coating, K is the strain optical coefficient of the coating and f is the fringe value of coating.

The advantage of the PhotoStress method is its high sensitivity for small strain/stress levels. On the other hand, in the areas with high strain/stress gradient, a problem can occur by quantifying the values (for the higher-order fringe recognition, the microscope is required to be used).

### 3.2. Digital Image Correlation Method

**Figure 11:**
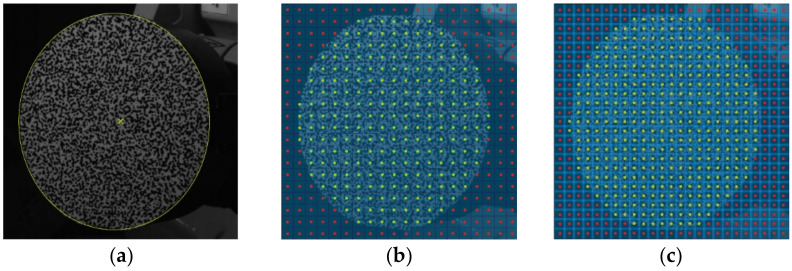
Illustrative example of the increase of the data resolution caused by the facet overlap: (**a**) defined area of interest; (**b**) measuring points (green dots) when the facets are in touch, (**c**) measuring points (green dots) when the facets are overlapped.

The deformation analysis was also carried out by a low-speed digital image correlation system Q-400 (Dantec Dynamics A/S, Skovlunde, Denmark) working on the principle of the DIC method (some detailed information are presented in Appendix B). The digital image correlation principle is based on the correlation of digital images captured during loading of the analyzed object, where the images are not compared as whole units, but as small image elements called facets. The shape of the facet used by correlation systems Dantec Dynamics is squared, and each facet commonly comprises a group of pixels ranging in size from 15 × 15 px to 30 × 30 px. Depending on the type of analysis, the facet size can be adjusted (reduced or enlarged). The facets may touch or overlap, but there must not be an empty area between them. Since the information about the displacements of the analyzed object is obtained at the nodes of the virtual grid, which correspond in position to the centers of the facets, the overlap of the facets (Figure 11) is one way to increase the data resolution, i.e., to obtain a more considerable amount of data and, thus, to better reconstruct the surface of the analyzed object (especially around the edges). The manufacturer of correlation devices, Dantec Dynamics, recommends overlapping the facets up to 1/3 of the facet size because with such an overlap the data points are still independent.

**Figure 12:**
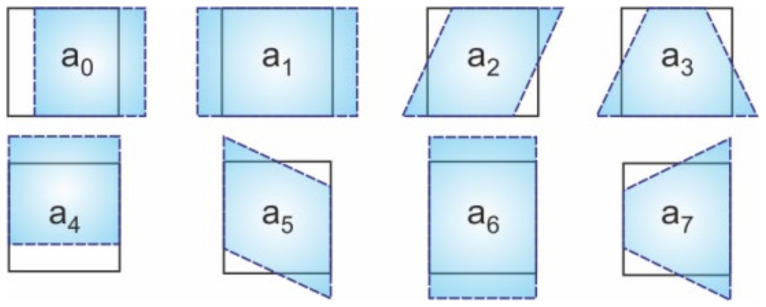
Transformation parameters used in the algorithm based on pseudo-affine transformation.

Dantec Dynamics correlation devices use an algorithm based on a pseudo-affine transformation to obtain information on the transformation coordinates of the analyzed object surface points. If transformation parameters of possible displacement, elongation, shear, and distortion of the facet $a_{0}-a_{7}$ as shown in Figure 12 are considered, using the aforementioned algorithm the transformation coordinates $\left(u,\;v\right)$ can be calculated as follows:

(2) $$\begin{array}{l} u\left(a_{0},a_{1},a_{2},a_{3},\tilde{x},\tilde{y}\right)=a_{0}+a_{1}\cdot \tilde{x}+a_{2}\cdot \tilde{y}+a_{3}\cdot \tilde{x}\cdot \tilde{y},\\ v\left(a_{4},a_{5},a_{6},a_{7},\tilde{x},\tilde{y}\right)=a_{4}+a_{5}\cdot \tilde{x}+a_{6}\cdot \tilde{y}+a_{7}\cdot \tilde{x}\cdot \tilde{y}, \end{array}$$

where ${\left(\tilde{x},\tilde{y}\right)}^{T}$ are the lens-distorted 2D coordinates of the point in the normalized image plane [31].

There are some relevant differences between the measurements of relieved deformations performed by 2D DIC and 3D DIC, respectively. Firstly, if the hole-drilling causes normal displacement of the analyzed specimen surface (this displacement component cannot be analyzed by a single-camera system), it leads to a correlation error and affects the analysis results. Another advantage of the 3D correlation system is the possibility to measure all the three displacement components not only of a flat but also curved specimen. Higher distortion of the lenses, which are not directed perpendicularly to the analyzed object surface, can be considered a small drawback of the 3D systems. Also, the requirement for a proper arrangement of the cameras towards the analyzed object means that the analysis performed by the 3D correlation system is done from a greater distance to the specimen. However, this limitation can be reduced using quality CCD cameras with sufficient image resolution of the sensors.

The accuracy of the results of the deformation analysis for both types of the analysis (2D or 3D) can be affected, for example, by:

- The quality of the speckle-pattern created on the analyzed object surface and illumination

**Figure 13:**
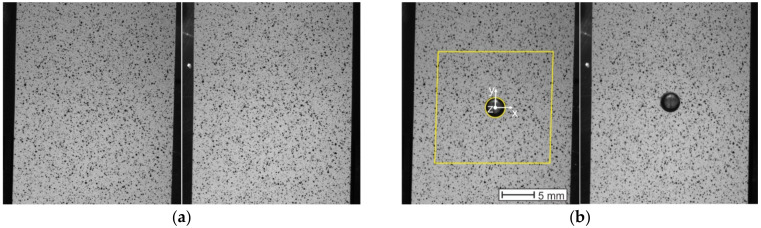
Pairs of digital images captured by 3D Q-400 Dantec Dynamics: (**a**) reference image; (**b**) evaluated image with a defined mask of evaluation.

For correct image correlation, it is essential to make sure that each facet is unique, i.e., it contains a unique pixel distribution with different levels of intensity of grey color. As the aim of the analysis was to use image resolution of the sensors as well as possible and to perform evaluation of the results near the edge of the drilled hole, it was necessary to create the black-and-white speckle-pattern with very fine speckles (Figure 13). The illumination of the specimen surface should be homogenous and reach high sharpness and sufficient contrast of speckles. Compliance with this requirement was ensured using the Dedolight DLH400DT (Dedo Weigert Film GmbH, München, Germany) halogen reflector with white light.

- Calibration parameters

The correctness of the coordinates transformation of the object points from 3D world coordinate system into 2D sensor system depends on the accuracy of the obtained external (i.e., mutual position and rotation of the cameras) as well as internal (focal length, principal point coordinates, tangential and radial distortion of the lenses) calibration parameters of the cameras. The calibration of Dantec Dynamics correlation systems is automatized, and based on the Zhang algorithm [32]. Information about the calibration accuracy is provided by the so-called calibration residuum, whose value should not exceed 0.5 px. The size of the calibration target should approximately correspond to the size of the analyzed specimen. Therefore, calibration of the stereo-camera correlation system was done using a calibration target comprising 9 × 9 checkboard fields with a precisely defined distance of 3 mm. The residuum obtained by the cameras calibration reached the value of 0.279 px, which is, according to the aforementioned criterion, considered as accurate calibration.

- Correlation parameters and the related levels of smoothing

Both Q-400 Dantec Dynamics cameras captured the digital images with image resolution of 1800 × 2056 px. The pixel density was approximately 64 px/mm. During the analysis a couple of reference images (Figure 13a) captured by the maximum loading force of 250 N were taken in the Istra4D ver. 4.3.0 control software. The pair of images capturing the deformation of the analyzed specimen surface area after drilling of the hole (Figure 13b), was correlated with the reference one according to the following aspects. The facet size as one of the correlation parameters set up by the evaluation of the measurement was adapted to the fact that the drilled hole’s edge would be reconstructed as accurately as possible if the facet was as small as possible. However, facets are required to be unique, i.e., all the facets have to comprise randomly distributed pixels with a high range of gray values. Therefore, the measurement was evaluated with the facet size set to 23 × 23 px, and their overlapping of 6 px ensured the increase of the data points (points in which the displacements and strains were evaluated) resolution.

**Figure 14:**
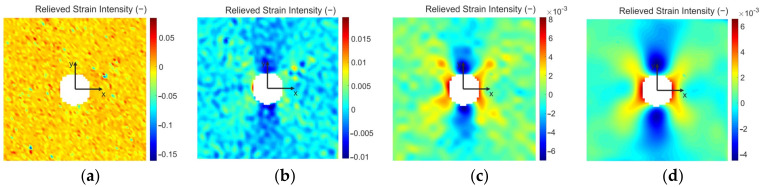
Influence of smoothing on the strain intensity field obtained by Q-400: (**a**) without loading; (**b**) kernel size of 7 × 7; (**c**) kernel size of 15 × 15; (**d**) kernel size of 31 × 31.

The results of the deformation analysis performed by the Dantec Dynamics correlation systems are due largely to the properly set smoothing level. Istra4D ver. 4.3.0 contains two types of smoothing. The first known as *local regression* should be used mainly in cases if a high deformation gradient is expected in the deformation analysis results. The second is called *smoothing spline* and is used to smooth the deformation field with approximately homogeneously distributed deformation levels. As with the hole-drilling method the stress concentrator occurs in the specimen, the authors used a filter of local regression. Several studies described, e.g., in [33,34] were conducted on the proper setting of the local regression level. For the illustration, the authors point to the effect of local regression in Figure 14, which shows the relieved strain intensity fields obtained by different levels of smoothing. Figure 14a shows the relieved strain intensity field obtained by default settings of smoothing, i.e., without smoothing. The other three relieved strain intensity fields correspond to the results obtained by the settings of kernel size to 7 × 7 (Figure 14b), 15 × 15 (Figure 14c), and 31 × 31 (Figure 14d).

According to the manufacturers of Dantec Dynamics correlation systems, the strains are computed only from the local curvatures of facets when the kernel size is set to 3 × 3. The higher the kernel size, the higher the influence of the deformation gradient on the strains. For the kernel size set to 9 × 9 up to 31 × 31 (i.e., the highest level of smoothing based on local regression in Istra4D ver. 4.3.0), the strains are computed only from the deformation gradients. According to the authors’ analysis, the kernel size of 15 × 15 for which the results obtained are presented in Figure 14c corresponds to the optimal setting of smoothing for the described type of evaluation. The effect of oversize smoothing can be observed in Figure 14d.

## 4. Results

Although the aforementioned measurement was conducted by two different non-contact optical methods providing the full-field information about the strains, the quantitative comparison of the results obtained from both techniques is not simple. While the results of the measurement by the DIC method provide quantitative information about the displacements as well as strains in the center of each facet, for the quantification of the results obtained using PhotoStress method, the use of a digital compensator is necessarily required in each evaluated location of the specimen.

In many cases, experimental testing results serve to verify the results obtained numerically, e.g., using software based on the finite element method (FEM) [35]. The above-described strain analysis in the vicinity of the hole drilled in the flat specimen loaded by uniaxial tension, is a typical analysis performed by the finite element analysis (FEA). For that reason, the authors reversed the standard approach and, thus, verified the experimentally obtained results by numerical analysis. Such an approach has already been used by the authors, and the results obtained were published in several scientific publications, e.g., [36,37,38,39]. Moreover, in conventional quantification of residual stresses by the hole-drilling technique the FEA is usually used, e.g., the correlation parameters $\bar{a},\;\bar{b}$ used in the formulas for the computation of stress components from the relieved strains are determined mainly in a numerical way.

### 4.1. Finite Element Analysis

The numerical model of the analyzed specimen was created in Abaqus/CAE 2020 (SIMULIA, Johnston, RI, USA) software. The analysis was focused on the determination of the relieved displacements/strains occurring in the specimen (with dimensions, mechanical properties, constraints and loading described in head 3) after milling a through-hole lying on its longitudinal axis. Detailed information on the procedure of numerical analysis is presented in Appendix C.

### 4.2. PhotoStress Method

**Figure 15:**
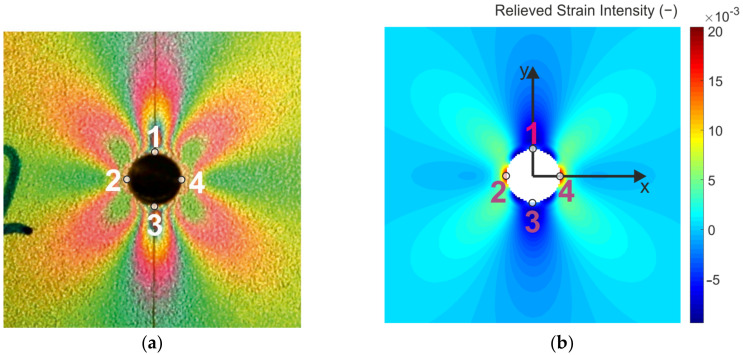
Relieved strain intensity analysis: (**a**) field of isochromatic fringes (PhotoStress); (**b**) finite element analysis (FEA).

The relieved strain analysis performed in the near vicinity of the cut through-hole was performed by studying isochromatic fringes distribution observed on the specimen loaded by uniaxial tension. The isochromatic fringes captured after the milling of the through-hole lying on the longitudinal axis of the specimen loaded by the tension force of 250 N are shown in Figure 15a.

Validation of the experimentally obtained results was done by comparison of the isochromatic fringes (interpreting the principal strain difference/strain intensity) with the strain intensity field obtained in Matlab (Figure 15b) as a consequence of stress-relieving (caused by the milling of the through-hole). The results obtained by the numerical analysis are symmetrical along the longitudinal axis of the specimen. However, the numerical analysis is carried out on an ideal model and with ideal boundary conditions. By comparing the results, the influence of the accuracy of the drilled through-hole on the measured values can be confirmed.

**Figure 16:**
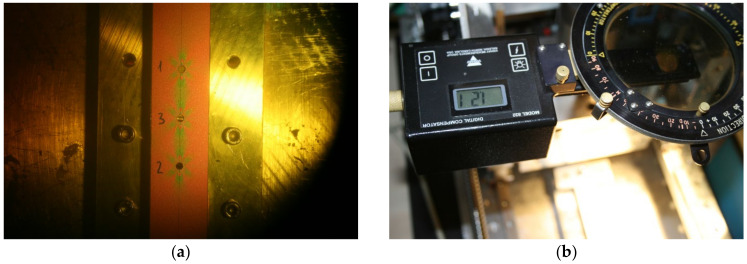
The use of the digital compensator: (**a**) isochromatic fringes observed after cutting the through-hole using the compensator; (**b**) fringe order assessment.

Four points (marked as 1, 2, 3 and 4) were chosen at the edge of the drilled through-hole (Figure 15a), in which the fringe order was assessed using digital compensator model 832 (Figure 16). Values of the relieved strain intensity in the aforementioned four points given in Table 3 were calculated according to Equation. (1).

As the process of fringe order assessment is not automatized, correct determination of the fringe order value is significantly dependent on the practical skills and experience of the experimenter. According to Table 3, it is evident that the differences in the obtained results are significant. As the highest gradient of relieved strain intensity occurs at 0.1D distance to the edge of the drilled through-hole (D = 3.2 mm is the diameter of the drilled through-hole), the difference in results can also be caused by the assessment of the fringe order near the edge of the hole. To obtain more precise results, it would be necessary to arrange the elements allowing micro-scaled measurement into the measuring device or to use the digital photoelasticity method, which results of application are described in detail, e.g., in the paper of Ramesh and Sasikumar [40]. As the authors’ workplace does not dispose of any of the aforementioned possibilities, the quantitative results obtained by the PhotoStress method need to be considered only as additional.

**Figure 17:**
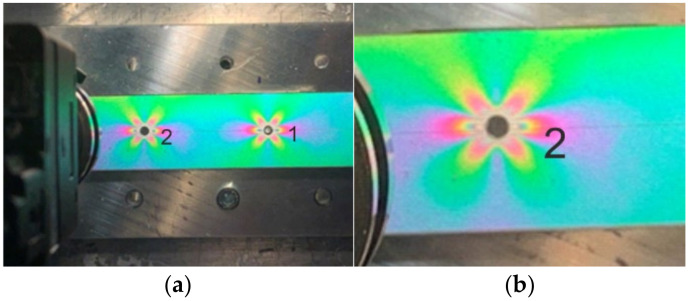
Demonstration of the PhotoStress method sensitivity—the through-hole denoted as 2 is not drilled at the axis of the specimen: (**a**) the overall view; (**b**) the detailed view.

The second (comparative) analysis in the surrounding of two other through-holes drilled to the same specimen loaded by the identical uniaxial loading was realized to qualify the sensitivity of the PhotoStress method. According to Figure 17, it is evident that the through-hole denoted as 1 was drilled on the longitudinal axis of the specimen loaded by the uniaxial tension. The center of the through-hole denoted as 2 was moderately biased from the axis of the specimen (see Figure 17b). At first glance, the isochromatic fringe patterns observed in the vicinity of both through-holes seem to be identical. The moderate differences in color patterns (mainly in areas near the edges) can be observed through a thorough visual analysis. In the vicinity of the through-holes, the differences are not significant.

Regular evaluation of the strain/stress analysis performed in the vicinity of the stress concentrator (close to of the drilled hole) is necessary to investigate residual stresses using the PhotoStress method. It can be stated that the results obtained from the optical method in combination with the hole-drilling method are directly dependent on the technical factors of both experimental techniques.

### 4.3. Digital Image Correlation (DIC) Method

**Figure 18:**
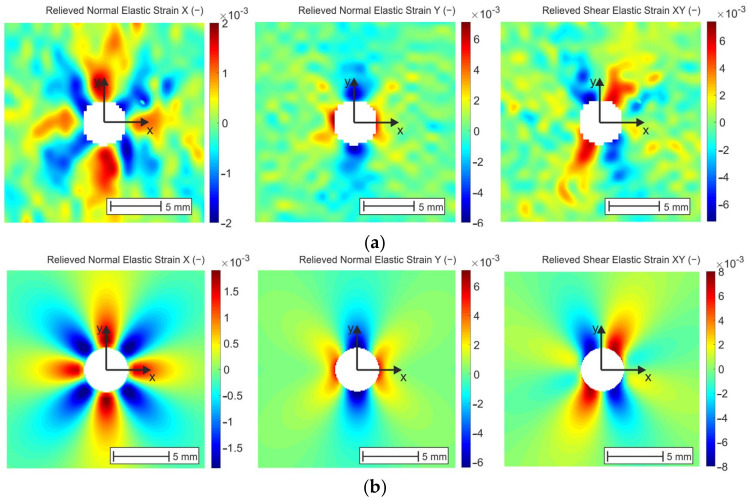
Qualitative comparison of the results obtained by (**a**) DIC; (**b**) FEA.

The relieved strain fields obtained from the analysis performed by Q-400 Dantec Dynamics and described in part 3.2 are shown in Figure 18a. Concerning the principle of Dantec Dynamics correlation systems, which evaluate the data at the centers of facets, it is not possible to reconstruct the contour of the drilled hole having a diameter 3.2 mm. The hole obtained by the correlation of the images using the correlation parameters described above is of a diameter of approximately 4 mm. To compare the results obtained experimentally and numerically both in qualitative and quantitative way, it was necessary to remove the corresponding elements lying on the circles concentric to the edge of the hole from the resulting strain fields and to visualize them again (Figure 18b). As can be seen in Figure 18, the strain fields’ distribution obtained by DIC corresponds with the results obtained numerically.

**Figure 19:**
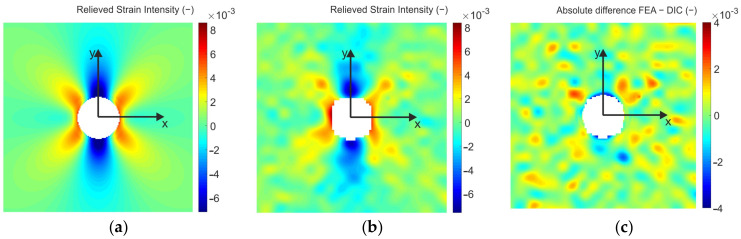
Relieved strain intensity fields obtained by: (**a**) FEA; (**b**) DIC; (**c**) absolute difference between the results obtained by FEA and DIC.

As the sensitivity of the DIC method is not as high as the PhotoStress method, the smaller the level of strain relieved is the higher differences in the results can be expected. In the case of small strains, as the drilled through-hole causes the stress concentration, it is convenient to perform the analysis as close as possible to the edge of the through-hole, where the highest levels of strains are relieved. On the other hand, the full-field comparison of the results obtained by FEA and DIC (Figure 19c), respectively, shows that the highest absolute difference between the results occurs just at the vicinity of the reconstructed through-hole edge.

**Figure 20:**
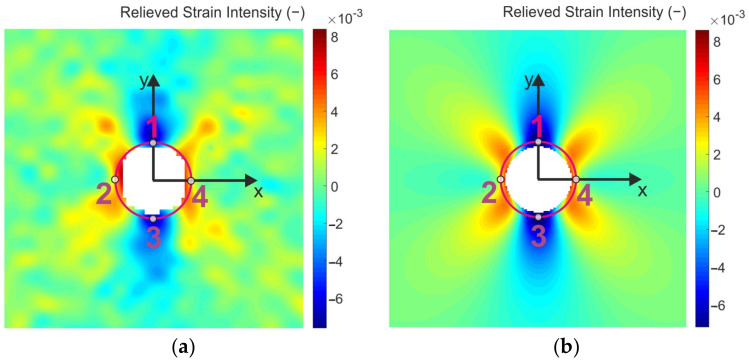
Location of 4 chosen (comparator) points and the comparison of the relieved strain intensity fields obtained by: (**a**) DIC, (**b**) FEA.

For the quantitative analysis of the results obtained by DIC and FEA, 4 points (marked as 1, 2, 3, and 4) lying on the circumference of the circle of radius 4.5 mm were chosen (Figure 20).

The strain intensity values obtained in selected points for 4 different levels of smoothing (see Figure 14) are listed in Table 4. As can be seen, the difference in the resulting values is significant. However, using the local regression smoothing with the kernel size set up to 15 × 15 (according to the previous investigations of the authors, such an adjusted level of smoothing should be the optimal one for the facet size of 23 × 23 px and their overlapping of 6 px) the relative differences between the results obtained in four selected points by FEA and DIC achieved values of approximately 14%.

**Figure 21:**
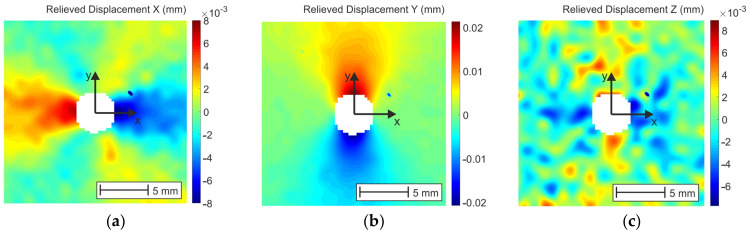
Relieved displacement fields obtained after filtering out the effect of the rigid-body motion: (**a**) displacement X, (**b**) displacement Y, (**c**) displacement Z.

Another possibility to minimize correlation errors is to take into account the displacement fields (Figure 21), which are not so significantly affected by noise occurring by small deformation levels as the strains. The possibility of filtering out the effect of rigid-body motion is also one of the advantages of work with the displacement fields of the DIC method. While 2D DIC allows obtaining two displacement components in the analyzed object surface plane, 3D DIC provides displacements in three mutually perpendicular directions. One of the methodologies for calculation of the residual stresses from the displacement fields was developed by Makino and Nelson in 1994 [41].

**Figure 22:**
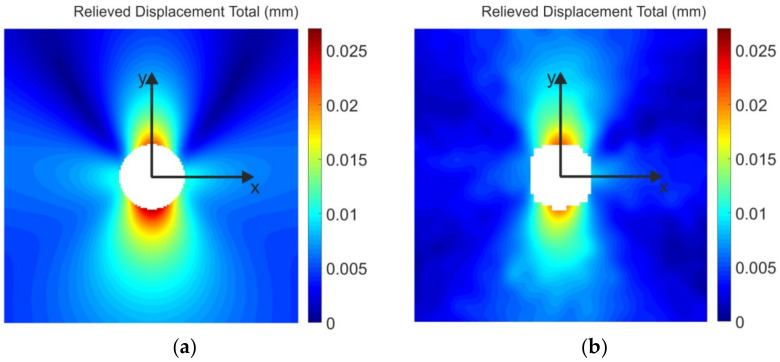
Relieved displacement total fields obtained by: (**a**) FEA, (**b**) DIC.

**Figure 23:**
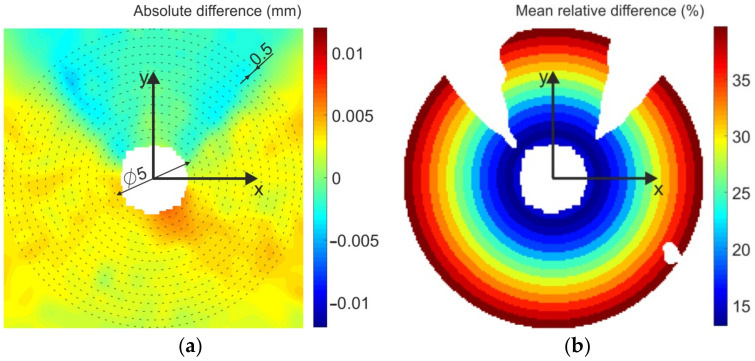
Difference between the relieved displacement total fields obtained by FEA and DIC: (**a**) absolute difference, (**b**) mean relative difference.

The full-field comparison of the results obtained by FEA and DIC was carried out on the relieved displacement total fields (Figure 22). Figure 23a shows their absolute difference field. As most of the developed methodologies are based on quantifying residual stresses from displacements, it was necessary to determine the relative deviation of the measured data from the reference data, i.e., FEA data. For that reason, the mean relative differences between the relieved total displacements obtained in points located at the area of concentric annuli of width 0.5 mm (see Figure 23a) were calculated as follows:

(3) $${diff}_{relative}^{mean}=mean\left(abs\left(\frac{{dispT}_{FEA}\left(i,j\right)-{dispT}_{DIC}\left(i,j\right)}{{dispT}_{FEA}\left(i,j\right)}\right)\cdot 100\%\right)$$

where i,j are the coordinates of the corresponding points, in which the data were compared.

To avoid the results being influenced, the significant relative difference occurred (mainly in two areas, where the results obtained by FEA approached zero) were removed from the calculation (see the empty areas in Figure 23b). It can be stated that the relative difference increases with the distance from the center of the cut through-hole. However, for the area of diameter approximately 9 mm, the maximum relative difference was 21% corresponding to the accuracy of DIC achieved by residual stress quantification [42].

**Figure 24:**
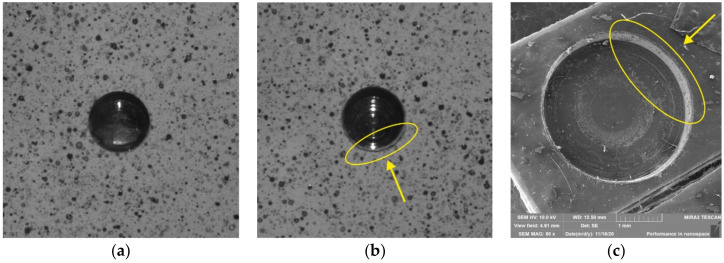
Milling the through-hole to the coating: (**a**) correctly milled through-hole without damage of the speckle-pattern; (**b**) damage of the speckle-pattern near the edge of the milled through-hole caused by the worn cutting miller; (**c**) analysis of the through-hole surrounding using an electron microscope.

In some measurements (mainly by the repeated use of the milling cutter) performed by the authors, a small part of the speckle-pattern located near the edge of the drilled through-hole was corrupted (see Figure 24).

As this phenomenon was not visible with the naked eye (the authors observed it only in the digital images obtained by the DIC system), the cause was analyzed using scanning electron microscope FE SEM MIRA 3 (TESCAN, Brno, Czech) by 60× magnification (Figure 24c). It was found that the worn cutting miller caused small indents at the edge of the through-hole cut into the coating made from PS-1D material and damaged the background (white) color layers. Correlating such problem areas can lead to correlation problems/errors. For this case, the authors recommend that the evaluation mask is defined outside the damaged area.

## 5. Discussion

In this paper, the development of the device designed for the strain and stress analysis performed on the specimen made from various kinds of material (e.g., plastics, metals, composites, etc.) using the hole-drilling technique and combining the full-field optical methods (PhotoStress, DIC) is described. As the technique of digital image correlation is based on the correlation of digital images captured during the measurement process, high positioning accuracy is required. Through the experimental testing, the measurement series were performed and showed the following results:

- positioning accuracy reached like 10^−3^ mm in both horizontal and vertical direction,
- high repeatability of the measurement comparable to the repeatability obtained with top commercially produced drilling devices (see Figure 5).

As most of the authors do not provide any detailed information about their devices, to compare our device with the devices developed and described in the introduction to this paper is a relatively complex issue. Despite it, the pros and cons of the designed device can be mentioned. The main pros are:

- the measurement realized using two optical systems (based on PhotoStress and DIC method) simultaneously, which can lead not only to the verification of the results but also to further improvements of methodology for the residual stresses quantification;
- quick and accurate change of the DIC system position towards the analyzed specimen allowing capturing of the images from the desired distance;
- the possibility to analyze and compare the results obtained by using single- and stereo-camera DIC system, which can improve the methodology for the realization of measurements by the DIC method.

The dimensions of the designed device can be considered as its disadvantage. Compared with the commercially produced devices, which have been developed and optimized for many years, our prototype is more massive and, thus, portable and manipulable with more difficulty. The reason for this is that our drilling device’s design has considered the minimum required dimensions of the working space. The measuring systems (single- or stereo DIC system, PhotoStress—reflective polariscope) work on optical principles and, thus, the analyzed specimen needs to be illuminated properly. The proposed prototype of the hole-drilling device was optimized so that the negative influence of the mechanical clearances was eliminated. In the future, the optimization of its dimensions in terms of weight minimization (by maintaining the sufficient stiffness requirement) is planned. To minimize the other components (servomotors, linear guides, etc.), it is necessary to consider their utility by operating load.

The methodology of evaluating the results in the experimental part by comparing the relieved strain fields obtained by the optical methods and numerical modeling is described in the second part of the paper. According to the results obtained, it can be stated that the PhotoStress method provides immediate information about the strain distribution with the sensitivity similar the other interference-based full-field methods, e.g., ESPI and moiré interferometry that allows its using also for the quantification of the residual stresses of smaller magnitudes. Comparison of the relieved strain intensity fields obtained by FEA and the PhotoStress method showed that using this optical method for full-field qualitative analysis is possible. However, the quantitative analysis carried out in four selected points located at the edge of the drilled through-hole suggests the need for farther improvement of the measurement methodology. To eliminate the measurement error, the authors plan to perform the sensitivity analysis of the results obtained in the area located close to the edge of a drilled hole.

The results obtained from the digital image correlation method are in the form of displacement and strain fields. The software Istra4D ver. 4.3.0 provided with the Q-400 correlation system Dantec Dynamics allows correlation parameters to be set up, i.e., facet size and overlapping, which need to be adapted to the size of speckles created on the analyzed specimen surface. According to the manufacturer, the strains are calculated from the local curvatures of the facets or deformation gradient depending on the smoothing adjustment level. In the paper, the effect of various levels of local regression smoothing is presented (Figure 13). With default settings, i.e., without smoothing, the results obtained in four selected points compared with numerical ones differ by approximately 400%. However, with the optimal level of smoothing determined by the authors the results differ by approximately 14% (Table 4). It has to be noted that the aforementioned relative difference cannot be achieved in the entire strain field, but only in the locations of such strain levels, which can be evaluated by the correlation system with sufficient accuracy. For that reason, it is convenient to work with the displacement fields, which are not as influenced by the smoothing used. A full-field comparison of relative differences between the total displacement fields obtained by FEA and DIC shows that the highest accuracy of the results is achieved in the vicinity of the cut through-hole (approximately to the 5 mm distance from the center of the hole). In such a case, DIC results differ from the results obtained numerically maximally by 20%, which is the expected accuracy of DIC in residual stresses analysis.

There is no known universal procedure applicable to set up all the evaluating software parameters used to determine stress components from strains/displacements. For that reason, before the analysis is made on the real structures, it is convenient to take measurements in the laboratory. To realize a series of laboratory measurements leading to the optimization of the methodology for quantifying residual stresses determined from the strains obtained by the PhotoStress method or by displacements/strains evaluated by the DIC method is one of the authors’ future aims. For their validation, the commercially produced devices RS200 and SINT MTS 3000 will be used, with which the authors have a lot of experience gained by solving technical problems in practice. Finally, the authors plan to use the developed prototype for the residual stresses analysis outside the laboratory.

## Figures and Tables

**Table 1:** Displacement resolution and disadvantages of the optical methods described.

Method	Displacement Resolution	Disadvantages
MI	10 nm with a pitch of 1 μm and a phase resolution of 2π/100	Requirement for a creation/installation of a grating and interferometric stability
ESPI	Typically <50 nm depending on the laser wavelength and the geometrical arrangement	Requirement for an interferometric stability
DIC	±0.01–0.02 of a pixel for the in-plane components, *Z*/50,000 * for the out-of-plane component	Requirement for creation of very fine speckles

* Note: *Z* is the distance from the object to the camera for a typical stereo-camera system.

**Table 2:** Experimentally determined values of depths and diameters of the blind holes drilled by the designed prototype of the drilling device.

Location	Milling Cutter	Cutting Speed (rpm)	Average Value of the Blind Hole Diameter		Average Value of the Blind Hole Depth *		
			Direction X (mm)	Direction Y (mm)	HBM (mm)	ASIMETO (mm)	Microscope (mm)
1	F1/S1	6000	3.271	3.243	1.987	1.978	1.995
2	F1/S1	6000	3.276	3.250	1.995	1.990	1.994
3	F1/S1	6000	3.235	3.255	2.009	2.015	2.006
4	F2/S1	10,000	3.274	3.281	2.000	1.995	2.004
5	F2/S1	10,000	3.257	3.257	1.989	1.991	2.003
6	F2/S1	10,000	3.252	3.259	1.998	2.003	2.000
7	F2/S2	10,000	3.200	3.203	2.005	1.993	2.007
8	F2/S2	10,000	3.202	3.202	2.001	2.004	2.004
9	F2/S2	10,000	3.200	3.202	2.004	2.001	2.003
10	F2/S2	6000	3.201	3.205	2.006	2.006	2.005
11	F2/S2	6000	3.202	3.199	2.021	2.014	2.003
12	F2/S2	6000	3.199	3.201	2.010	2.008	2.001
13	F3/S1	6000	3.245	3.246	1.999	1.998	2.000
14	F3/S1	6000	3.249	3.225	2.001	1.996	2.001
15	F3/S1	6000	3.251	3.246	2.003	2.010	1.993
16	F3/S1	10,000	3.242	3.255	2.011	2.015	2.003
17	F3/S1	10,000	3.243	3.239	2.008	2.007	2.005
18	F3/S1	10,000	3.214	3.238	2.013	2.004	2.001
19	F3/S2	6000	3.257	3.245	2.006	2.008	2.006
20	F3/S2	6000	3.242	3.240	2.008	2.001	2.000
21	F3/S2	6000	3.237	3.252	2.012	2.020	1.999
22	F3/S2	10,000	3.252	3.255	2.005	2.011	1.999
23	F3/S2	10,000	3.251	3.255	2.007	2.009	2.003
24	F3/S2	10,000	3.243	3.264	2.011	2.010	2.002
AV **	-	-	3.237	3.238	2.004	2.004	2.002
SD ***	-	-	0.0145	0.0130	0.0013	0.0021	0.0003

* Note: The final depth of the drilled blind hole adjusted by the control software was 2 mm. ** AV—Average value (mm), *** SD—Standard deviation (mm).

**Table 3:** Comparison of the values of relieved strain intensity obtained in four selected points located at the circumference of the drilled through-hole with a diameter of 3.2 mm.

Point	PhotoStress		FEA
	Fringe Order	ε_1_ − ε_3_	ε_1_ − ε_3_
1	1.21	−0.00459	−0.00112
2	3.26	0.01235	0.02039
3	1.20	−0.00455	−0.00112
4	3.01	0.01141	0.0204

**Table 4:** Comparison of the relieved strain intensity obtained in four chosen points using DIC by 4 different levels of smoothing and FEA.

Relieved Strain Intensity					
Point	DIC				FEA
	Without Smoothing	Kernel Size 7 × 7	Kernel Size 15 × 15	Kernel Size 31 × 31	
1	−0.01095	−0,00734	−0.00685	−0.00296	−0.00793
2	0.00327	−0.00058	0.00347	0.00374	0.00307
3	−0.00394	−0.00831	−0.00692	−0.00253	0.00793
4	0.00639	0.00089	0.00326	0.00327	0.00307

**Figure A1:**
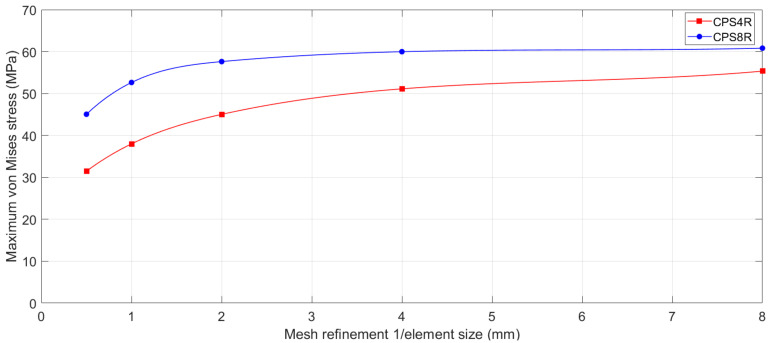
Convergence analysis of the solutions using two types of finite elements (CPS4R and CPS8R).

**Figure A2:**
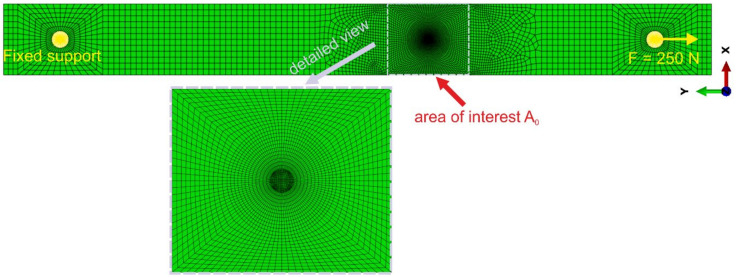
Meshed model and boundary conditions of the specimen without the net cross-section; detailed view on the finer mesh created in the area of interest.

**Figure A3:**
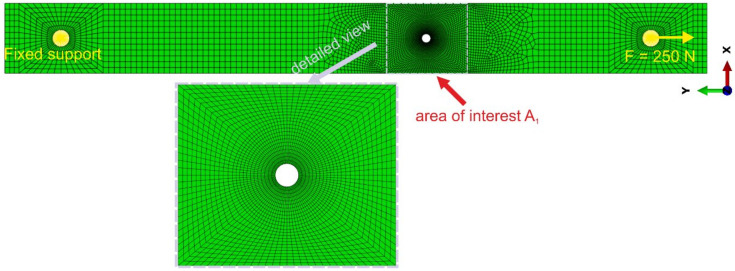
Meshed model and boundary conditions of the specimen with the through-hole created in the area of interest; detailed view on the finer mesh created in the area of interest.

**Figure A4:**
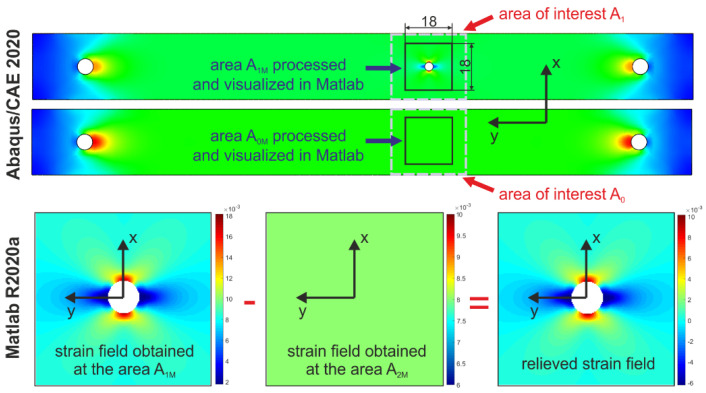
Schematic visualization of strains relieved determination using FEA performed in Abaqus/CAE 2020 and post-processing of the exported data in Matlab R2020a.

## Data Availability

Data sharing is not applicable to this article.

## Appendix A

1. Compute the combination strains p, q and t for the relieved strains $\varepsilon_{a}$, $\varepsilon_{b}$ and $\varepsilon_{c}$ measured by strain gauge rosette using:
  (A1) $$\begin{array}{l} p=\frac{\varepsilon_{c}+\varepsilon_{a}}{2},\\ q=\frac{\varepsilon_{c}-\varepsilon_{a}}{2},\\ t=\frac{\varepsilon_{c}+\varepsilon_{a}-2\cdot \varepsilon_{b}}{2}\;. \end{array}$$
2. Determine the calibration constants values $\bar{a},\;\bar{b}$ corresponding to the hole diameter and type of strain gauge rosette used. Compute the three combination stresses P, Q and T corresponding to the three combination strains p, q and t using:
  (A2) $$\begin{array}{l} P=\frac{\sigma_{y}+\sigma_{x}}{2}=-\frac{E\cdot p}{\bar{a}\cdot \left(1+\mu \right)},\\ Q=\frac{\sigma_{y}-\sigma_{x}}{2}=-\frac{E\cdot q}{\bar{b}},\\ T=\tau_{xy}=-\frac{E\cdot t}{\bar{b}}, \end{array}$$
  where P = isotropic (equi-biaxial) stress, Q = 45° shear stress, T = xy shear stress. The calibration parameters are determined either by experimental investigation of the calibration specimen or using numerical modeling.
3. Compute the principal stresses $\sigma_{\max}$ and $\sigma_{\min}$ using:
  (A3) $$\sigma_{\max},\;\sigma_{\min}=P\pm \sqrt{Q^{2}+T^{2}}.$$

## Appendix B

The 3D measuring system used for the analysis consists of two Allied Stingray F504G cameras with a CCD sensor to capture images in maximum 5 Mpx resolution (2452 (H) × 2056 (V) px). The process of camera calibration, measurement as well as the evaluation was performed in Istra4D ver. 4.3.0 control software, provided with correlation systems Dantec Dynamics. Other technical parameters of the Q-400 correlation system are given in Table A1.

**Table A1:** Technical parameters of the Q-400 Dantec Dynamics system.

Measurement Area	Adjustable: from mm^2^ to m^2^
Measuring range	displacements: approximately 10^−5^ of the field of view, which corresponds to 1 μm at a field size of 100 mm^2^, strains: from 0.01%—several 100%
Image resolution	max. 2452 × 2056 px up to 9 fps
Measurement results	the spatial contour of the object surface, 3D displacements, strains at each point
Control and monitoring electronics	portable laptop with Windows 7, 16-bit analog-to-digital converter (8 channels with a voltage range of ±0.05 V to ±10 V)
Lighting	halogen reflector with white light

## Appendix C

The numerical determination of strains relieved is divided into several steps:

1. Creation of the analyzed specimen model (with dimensions according to Figure 9a) without the net cross-section in the specimen’s analyzed area.

Note: the holes were created in the ending part of the model (at sufficient distance to the area of interest) for definition of boundary conditions, i.e., loading tension force of 250 N, and fixed support simulated the constrain of the specimen used in experimental analysis.

2. Convergence analysis of the solutions (Figure A1) performed by the stress analysis of the model with the net cross-section using different types of elements (e.g., CPS4R and CPS8R) with varying mesh refinement (e.g., 1/element size = 2, 1, 0.5, 0.25, 0.125, etc.). Selection of the proper element type (CPS8R) and sizing (0.5 mm) in the area of interest.

Note: the through-hole in the area of interest is created by removing of the finite elements which locations correspond to the hole with a diameter of 3.2 mm.

3. Creation of the finite element mesh using the linear quadrilateral elements of type CPS8R and its sizing (0.5 mm) defined in the analyzed area of interest A_0_ (with the size of 25 (X) × 30 (Y) mm^2^) with the total number of finite elements creating the area of interest 3560, and the number of nodes 10791 (Figure A2); definition of boundary conditions and material properties.

4. Realization of the strain analysis on the specimen without the net cross-section; export data from the nodes located in the area of interest.
5. Realization of the strain analysis on the specimen with the cut through-hole (Figure A3); export data from the nodes located in the area of interest.

Note: the finite element model with the area of interest A1 (size of 25 (X) × 30 (Y) mm^2^) was obtained, where the elements and the nodes have the same locations as in the case of the model without the net of the cross-section.

6. Import data into the software Matlab R2020a (Mathworks, USA), the creation of a new mesh, assignment of the data values imported from the FE model nodes to the corresponding locations on the mesh created in Matlab.
7. Computation of the relieved strains as a difference in particular data (normal elastic strain X, normal elastic strain Y, tangential shear strain XY, strain intensity) values obtained in the areas of interest A1 and A0, respectively, their visualization for the squared areas A1M and A0M of 18×18 mm^2^ (Figure A4).
